# Analysis of the application values of different combination schemes of liquid-based cytology and high-risk human papilloma virus test in the screening of high-grade cervical lesions

**DOI:** 10.1590/1414-431X20187517

**Published:** 2018-11-23

**Authors:** Jian Wang

**Affiliations:** Department of Obstetrics and Gynecology, Institution Zaozhuang Municipal Hospital, Zaozhuang, Shandong Province, China

**Keywords:** Liquid-based cytology, Human papilloma virus, Cervical cancer, ROC curve, High-grade

## Abstract

The aim of this study was to explore the value of different combination schemes of liquid-based cytology (LBC) and high-risk human papilloma virus (HPV) test in the screening of high-grade (≥CIN 2) cervical lesions. From 5727 women who had undergone examinations with LBC and high-risk HPV test, 1884 patients with positive results of either or both LBC and HPV test were included in this study and underwent cervical biopsy. Based on the pathological examination results, comparisons of the assessment indicators of all diagnostic tests were made, and the application values of LBC and high-risk HPV test and different combination schemes of the two in the screening of high-grade (≥CIN II) cervical lesions were estimated. Compared with the single test method, the sensitivity and negative predictive value of the combination scheme of LBC+HPV (with one positive result) were increased significantly (98.7% and 99.7%), but the specificity (60.8%) and accuracy rate (65.4%) dropped significantly (P<0.05). The sensitivity of LBC+HPV (with two positive results) was the lowest (80.7%), but the specificity and accuracy rate were the highest (83.5% and 83.1%, P<0.05). Z test showed that differences in the screening efficiency of four schemes were not statistically significant (P>0.05). Both LBC and HPV test were effective methods in the screening of high-grade cervical lesions; combination of the two tests did not improve the screening efficiency, but the scheme of LBC+HPV (with two positive results) significantly increased the sensitivity and negative predictive value, which was of better cost-benefit value.

## Introduction

Cervical cancer is the most common malignancy in female reproductive system, which seriously threatens women's health. Deaths due to cervical cancer reach approximately 275,000 every year (1). It takes approximately 8 to 12 years for the cervical intraepithelial neoplasia (CIN) and pre-invasive carcinoma to progress into invasive carcinoma (2,3). Therefore, early discovery and treatment of precancerous cervical lesions are especially important in the prevention of cervical cancer.

Studies show that persistent infection of high-risk human papilloma virus (HPV) is the main reason of cervical cancer and precancerous lesions (4–6). The positive rate of HPV in cervical cancer tissue samples was 99.7% (7). The HPV genome is a circular double-stranded DNA containing nearly 8,000 base pairs (bp) and consists of three parts, including the early region (E), late regions, and long control regions. The early region containing E1, E2, E4, E5, E6, E7, and a total of 6 genes that maintain viral replication, encode viral proteins and maintain high copy number of intracellular viruses.

At present, the HPV test is becoming an important method in the screening of cervical cancer, but the specificity of the test in detecting CIN 2/3 is slightly lower when compared with cytology (8). Liquid-based cytology test (LBC) significantly improves the detection rate of cervical lesions (9), but the problem is the existence of false negative results (10). Therefore, several institutions including the American Cancer Society recommended the combination of LBC and HPV tests in the screening of cervical cancer (11,12). In China, LBC and high-risk HPV test are the two chief methods among various screening methods, but reports on the two methods in China and the clinical assessment indicators of combination schemes of the two are relatively few, and the evaluations and conclusions of the diagnostic values acquired by different laboratories are not the same (13).

This study provided the basis for scheme selection in screening cervical cancer through a comprehensive evaluation of the diagnostic value of LBC, HPV, and different combination schemes of the two for precancerous cervical lesions.

## Material and Methods

### Study subjects and study design

A total of 5727 patients in Zaozhuang Municipal Hospital were collected from January 2012 to December 2012. Patients enrolled in the study had a normal sexual life, had not undergone hysterectomy, cervical conization or pelvic radiotherapy, had not been diagnosed with CIN, were not pregnant, were not in their menstrual period, did not use steroid hormones in the past three months, did not have sex in the past 48 h, did not receive vaginal irrigation or any other local treatments.

Women included in this study had suspected cervical disorders or positive results of either or both cytology and HPV, and underwent colposcopy and cervical biopsy in the past two months. Pathological results verified different degrees of cervical lesions. Finally, a total of 1884 patients aged 17 to 71 years (average age, 33.5±8.1) were included. For the sample collection and treatments, written informed consent was provided.

### Study methods

#### Liquid-based cytology (LBC)

Samples were collected using the Cervex Brush (Rovers Medical Devices, B.V. Oss, Netherlands) according to the product instructions. The brush was rotated three to five times at the endocervix, and then the brush with the collected sample was directly immersed in the SurePath preservative fluid. The slides for LBC were prepared by the fully automatic liquid-based cytology system from BD Company (USA). Then the cytology smears were examined by experienced senior cytologists who were not aware of the HPV test results. Diagnoses were made according to the Bethesda System classification criteria. LBC (+) was defined as the diagnosis of atypical squamous and glandular cells of undetermined significance (ASC-US).

#### DNA extraction method

We added 1 mL of double distilled water into a 1.5 mL centrifuge tube containing cervical exfoliated cells. The tubes were vigorously shaken (3000 rpm) for 10 min, centrifuged at 19,942 *g* for 5 min at 20^o^C, and the supernatant was discarded (the blood sample was repeated once). We added 100 μL of HPV-DNA extract to the cell pellet, mixed well, and boiled at 100°C for 10 min. The tubes were then centrifuged at 19,942 *g* for 5 min at 20^o^C.

#### HPV test

Following LBC, the remaining samples were used for HPV typing; the typing kits were purchased from Cape Biotechnology Co. Ltd. (China). A total of 15 high-risk types (16, 18, 31, 33, 35, 39, 45, 51, 52, 53, 56, 58, 59, 66, 68) and 6 low-risk types (6, 11, 42, 43, 44, CP8304) were involved in the HPV test. Samples with high-risk HPV types were defined as HPV (+).

#### LBC+HPV tests

1) LBC+HPV (with one positive result), i.e., result of the combined scheme was determined as positive with one positive result of either test; 2) LBC+HPV (with two positive results), i.e., result of the combined scheme was determined as positive with two positive results of both tests and it was negative if one test result was negative.

#### Colposcopy and cervical biopsy

Patients with one or two positive results of LBC and HPV test, or with negative LBC and HPV test results but with cervical abnormalities (such as cervical ulceration, bleeding, positive acetic acid test result, or other suspicious lesions) underwent colposcopy and cervical biopsy. All tissue samples were sliced according to pathological procedures, and slices were read and diagnosed by two experienced pathologists. Pathologists who conducted the exam did not know the results of other tests.

### Statistical analysis

SPSS13.0 (IBM, USA) and MedCalc statistical software (Belgium) were used for analysis. Based on the golden standard of pathological results, χ^2^ test was used for comparison of the detection rates of different cervical lesions by the four schemes. Various assessment indicators of the four schemes in the screening of high-grade (≥CIN 2) cervical lesions were calculated, including sensitivity, specificity, accuracy, positive predictive value, negative predictive value, etc. Clinical values of the four schemes in the diagnosis of high grade (≥CIN 2) cervical lesions were assessed by comparing the areas under the receiver operating characteristic (ROC) curve.

## Results

### Detection rates of different cervical lesions by different test methods

A total of 620 patients were determined as LBC (+) (positive rate, 10.8%), and 1309 patients were determined as HPV (+) (positive rate, 22.9%). A total of 1884 patients had undergone the pathological examinations, and 694 patients were diagnosed with CIN (221 with CIN 2/3 and 12 with cervical cancer). Specific detection results of different cervical lesions by the four schemes consisting of the two methods are shown in Table 1. Positive detection rates of different schemes increased with the severity of cervical lesions; positive detection rates of the four schemes for the same grade lesion were significantly different (P<0.01). When subjects were tested by LBC or HPV alone, the positive detection rates of high-grade (≥CIN 2) cervical lesions all reached or were close to 90%, and it was above 97% for LBC+HPV (with one positive result). All differences were statistically significant (P<0.05).

**Table 1:** Detection rates of different cervical lesions by different test methods.

Results of histopathological confirmation	Cases	Test methods			
		LBC +	HPV +	LBC+HPV (one +)	LBC+HPV (both +)
Cervicitis or other benign lesion	1178	9.4 (111)	13.4 (158)	20.0 (236)	2.1 (25)
CIN1	473	64.9 (307)	71.9 (340)	87.1 (412)	52.4 (248)
CIN2	137	81.8 (112)	89.8 (123)	97.8 (134)	73.7 (101)
CIN3	84	92.9 (78)	95.2 (80)	100.0 (84)	89.3 (75)
Cervical carcinoma	12	100.0 (12)	100.0 (12)	100.0 (12)	100.0 (12)
Total	1884	32.9 (620)	37.8 (713)	46.6 (878)	24.5 (461)
χ^2^		865.12	886.15	900.31	984.91
P		0.000	0.000	0.000	0.000

Data are reported as percent with number of cases in parentheses. LBC: liquid-based cytology; HPV: human papilloma virus; CIN: cervical intraepithelial neoplasia.

#### Clinical assessments of different combination schemes

Sensitivity of LBC (86.7%) in the testing of high-grade (≥CIN2) cervical lesions was slightly lower than that of HPV test (92.3%), but the specificity and accuracy of LBC (74.7% and 76.2%) were higher than that of HPV test (69.8% and 72.6%), and the differences were statistically significant (P<0.05). The sensitivity and negative predictive value of LBC + HPV (with one positive result) reached 98.7% and 99.7%, respectively, which increased compared with LBC or HPV test alone. The specificity and accuracy dropped (P<0.05) while the result of LBC+HPV (with two positive results) were the opposite. Sensitivity and negative predictive value were lower (80.7% and 96.8%) but the specificity, accuracy, and positive predictive value (83.5, 83.1, and 40.8%) were higher than other schemes. All differences were statistically significant (P<0.05) (Table 2).

**Table 2:** Clinical assessments of different combination schemes.

Test methods	Sensitivity % (cases)	Specificity% (cases)	Diagnosis accuracy% (cases)	Positive predictive value% (cases)	Negative predictive value% (cases)	Area under ROC curve
LBC	86.7 (202/233)	74.7 (1 233/1651)	74.7 (1233/1651)	32.6 (202/620)	97.5 (1233/1264)	0.807
HPV	92.3 (215/233)*^#^	69.8 (1 153/1651)*^#^	72.6 (1368/1884)*^#^	30.2 (215/713)^#^	98.5 (1153/1171)^#^	0.810
LBC + HPV (one +)	98.7 (230/233)^&^	60.8 (1 003/1651)^&^	65.4 (1233/1884)^&^	26.2 (230/878)^&^	99.7 (1003/1006)^&^	0.797
LBC + HPV (both +)	80.7 (188/233)	83.5 (1 378/1651)	83.1 (1566/1884)	40.8 (188/461)	96.8 (1378/1423)	0.820

Data are reported as percent with number of cases in parentheses. LBC: liquid-based cytology; HPV: human papilloma virus. *P<0.05, HPV group compared with LBC group; ^#^P<0.05 HPV group compared with LBC+HPV (one +) group and LBC+HPV (both +) group; ^&^P<0.05 LBC+HPV (one +) group compared with LBC+HPV (both +) group.

### Comparison of the areas under ROC curve

According to the test results and the reference (14), ROC curves of the four schemes were drawn (≥ASC-US was used as the threshold value) and areas under the curves of the four schemes [LBC, HPV, LBC+HPV (with one positive result), LBC+HPV (with two positive results)] in the diagnosis of high-grade (≥CIN 2) cervical lesions were calculated; areas were 0.807, 0.810, 0.797 and 0.820, respectively. Differences of the detection efficacies of the four schemes were not significant by the *Z* test (P>0.05) (Table 3).

**Table 3:** Comparison of the area under ROC curve.

Items	AZ1-AZ2	Z value	P value
LBC *vs* HPV	−0.004	0.248	>0.05
LBC *vs* LBC + HPV (one +)	0.009	0.805	>0.05
LBC *vs* LBC + HPV (both +)	−0.014	1.606	>0.05
HPV *vs* LBC + HPV (one +)	0.013	1.487	>0.05
HPV vs LBC + HPV (both +)	−0.010	0.849	>0.05
LBC + HPV *vs* LBC + HPV (one +) (both +)	−0.023	1.690	>0.05

LBC: liquid-based cytology; HPV: human papilloma virus.

## Discussion

LBC and HPV tests are two main methods in the screening of cervical cancer, and the combination screening of the two methods is recommended by American Society for Colposcopy and Cervical Pathology (ASCCP) (15). In this study, the positive detection rates of high-grade (≥CIN 2) cervical lesions by LBC, HPV, and two combination schemes of LBC+HPV tests increased with the severity of cervical lesions, and were all above 80%. Positive detection rates of a same grade cervical lesion by different schemes were different. The detection rate of LBC was lower than that of HPV and LBC+HPV (with one positive result), and the detection rate of LBC+HPV (with two positive results) was comparatively low, which demonstrated that the four schemes were all effective in the screening of cervical cancer, but the detection rates of different grades of lesions by the four schemes were different.

High-risk HPV infection, commonly detected in women, was publically regarded as the pathogenic factor of cervical cancer (16,17). However, positive HPV alone does not represent the occurrence of cervical lesion. Positive LBC is found only when the morphological changes of cervical cells happens. Therefore, the positive detection rate of HPV is higher than that of LBC. Because the interpretation standard of LBC+HPV (with one positive result) was looser than that of LBC+HPV (with two positive results), the positive detection rate of the former was higher than that of the latter.

Positive detection rate is related to the sensitivity of screening schemes. Higher positive detection rate is followed by high sensitivity, but the false positive result rate is also probably high. Assessment of the diagnostic effectiveness of the screening method mainly relies on the evaluation results of diagnostic experiments (18,19). In this study, the sensitivity of HPV test was higher than that of LBC, but the specificity was lower than that of LBC, which showed that HPV test was better in the detection of cervical lesions, but the false positive rate might be even higher than that of LBC, which was in accordance with the current screening experiences both at home and abroad (20).

Combination screening method has been clinically recommended. This study compared the determination criteria of different combination schemes, and demonstrated that the sensitivity of LBC+HPV (with one positive result) in the screening of high-grade (≥CIN 2) cervical lesions reached 98.7%, but the specificity and accuracy of this scheme were lower than those of LBC or HPV tests alone. The sensitivity of LBC+HPV (with two positive results) was relatively low (80.7%), but the specificity and accuracy (83.5 and 83.1%) were the highest compared with other schemes, which was the same as a recent meta-analysis result (21).

Areas under ROC curves are regarded as an objective assessment of a diagnostic value of a test. Diagnostic values of different schemes can be evaluated by comparing the areas under ROC curves. Areas under ROC curve of LBC and HPV tests were 0.807 and 0.810, and there was no significant difference between detection efficiencies of the two in the screening of high-grade (≥CIN 2) cervical lesions, which showed that the diagnostic values of the two schemes in the screening of cervical cancer were equal. Areas under ROC curves of the two combination schemes were 0.797 and 0.820, and the difference between the two was not significant and the same compared with LBC or HPV tests alone, which demonstrated that the combination schemes cannot improve the screening efficiency significantly. The diagnostic values of the four schemes in the screening of high-grade (≥CIN 2) cervical lesions were equal, which was different from previous reports (13).

As for LBC or HPV tests alone, although there was no significant difference in screening efficiency, the sensitivity of HPV was higher than that of LBC and the missed diagnosis rate was relatively lower than that of LBC. A study showed that the risk of having CIN 3+ in the next three years for HPV-negative patient was significantly lower than that for LBC-negative patient (0.063 *vs* 0.17%), and the risk of having invasive cancer in the next five years for HPV-negative patient (0.17%) was only half of that for LBC-negative patient (0.36%) (11). It can be concluded that HPV-negative patients have a higher long-term predictive value than LBC-negative patients. Therefore, HPV test was more advantageous in the screening of cervical cancer.

It seemed that the combination schemes had no improvement in the screening efficiencies and the cost was increased, but the sensitivity of LBC+HPV (with one positive result) was the highest and the missed diagnosis rate was the lowest. From a clinical perspective, LBC+HPV (with one positive result) guaranteed early exposure of CIN lesions to the largest degree, which was beneficial to reduce the incidence of cervical cancer. Moreover, combination schemes had a higher negative predictive value (98.7%). A study showed that the risk of having CIN 3 in the next five years for females who had double negative results of LBC + HPV test was only 3.2/100,000 (females/year), and it was safe and effective to prolong the screening interval to five years for these females. The schemes were also recommended by ASCCP recently. The combination schemes provided a longer screening interval and reduced screening frequency, which decreased the screening cost with a long-term benefit.

There are some limitations for this study. Firstly, patients enrolled in this study had higher risk of cervical cancer than the general population, which made it unable to evaluate the overdiagnosis of cervical precancers or their progression probabilities. Besides, it is also difficult to accurately interpret the specificity parameters involved in this study in comparison with those for the general population. In addition, since there may be some variations between various test systems for HPV, it is better to conduct a systematical comparison between HPV test systems. The current HPV test system lacks systematical validation, which may discount the conclusion on HPV tests; therefore, the results can only be appropriate for the current HPV test system. Lastly, as HPV infections and CINs at young ages (<30 years old) will vanish naturally, further research about the value of different strategies across different age groups will be conducted in our future study.

In conclusion, LBC alone, HPV alone, and LBC+HPV combination schemes were all effective methods in the screening of cervical cancer. The scheme of LBC+HPV (with one positive result) may decrease the screening cost from a long-term perspective. Long-term systematic evaluations of the costs for this combination scheme are warranted. In clinical settings, with full consideration of medical conditions and screening costs, appropriate screening schemes should be chosen to improve the screening rate among high-risk populations.
